# The Contribution of Neuropsychoanalysis to Clinical Practice in Psychiatry

**DOI:** 10.62641/aep.v53i4.1953

**Published:** 2025-08-05

**Authors:** Tennyson Lee, Mathieu Norton-Poulin, Andrea Clarici

**Affiliations:** ^1^Deancross Personality Disorder Service, East London NHS Foundation Trust, E1 4DG London, UK; ^2^Gatineau Institute of Clinical Psychology, Gatineau, QC J8T 0B1, Canada; ^3^Department of Medical, Surgical and Health Sciences, Cattinara Hospital, University of Trieste, 34149 Trieste, Italy

Our patients usually present with unwanted feelings - ‘Dr. I’m feeling depressed/anxious, etc.’ Do we treat these feelings as psychological abnormalities needing to be extinguished or as indicators of underlying problems that need addressing? To help answer this, psychiatrists may benefit from developments in Affective Neuroscience.

One such development is Panksepp’s model of the brain, which describes seven basic emotional systems (BES) (see Table 1) [1] serving an evolutionary function in enhancing survival and reproduction [2]. This implies that not meeting these needs is counter to survival and can lead to common psychiatric symptoms e.g. panic followed by depression [3]. Affect arises as a signal that these BES needs are not being met [4]. Therefore, unwanted feelings will remain until the need is met. This clarifies the role of affect and fundamentally reframes how the clinician can view the patient’s complaint: ‘Dr. I’m feeling depressed/anxious, etc.’.

**Table 1.** **Panksepp’s Emotional Drives**.

SEEKING	Need to engage with the world to meet our biological needs. A ‘wanting’, foraging instinct. Felt as interest and curiosity.
LUST	Finding a sexual partner. Felt as sexual arousal.
CARE	Nurturing, e.g., of off-spring. Felt as “proto-empathy”. [5]
PANIC/GRIEF (linked to the attachment system)	We need to attach to those who look after us - separation from our attachment figures is felt as panic, and loss of them felt as *despair* (hence the link between anxiety disorder and depression). This system also links to Attachment theory. Note PANIC (wishing to get closer to) is not the same system as FEAR (wishing to get away from).
PLAY	This is more than (but does include) child’s play. This is how social hierarchies are formed and how ingroup and outgroup boundaries are maintained. Felt as “social joy”.
RAGE	Destroy or eliminate frustrating objects (i.e., obstacles to satisfying our needs). Felt as anger.
FEAR	Escaping from, or freezing within, a dangerous situation. Felt as anxiety.

A linked development is neuropsychoanalysis [6], which takes a neurobiological approach to how ‘we feel, behave, and think’. It integrates Panksepp’s BES, Freud’s theory of the mind, and Friston’s computational psychiatry [7] to indicate how neural circuits for basic emotions can influence complex psychological experiences and behaviours.

Solms [8] summarised the core scientific claims of Neuropsychoanalysis as follows:

1. We are born with innate needs and basic instincts that are designed to ensure survival and reproduction. When activated, these needs lead to reflexive (not reflective) behaviours. The affects linked to these behaviours are either positively or negatively valenced (affects are a “value” system), depending on whether we satisfy our basic emotional needs: i.e., affects are at the service of a homeostatic function and we strive to remain within, or to return to our set point (e.g., regarding the PANIC/GRIEF system, the set point is to have the attachment object close at hand and our behaviours are then directed at getting us to that point). Emotions, rather than cognition, are the primary drivers of our motivated behaviours.

2. The main task of psychological development is to learn how to fulfil our needs within our environment and eventually in the most economic, automatized way. This is primarily the function of implicit memory: storing sequences of behaviours that satisfy our (often conflicting) needs. These behaviours, i.e., predictions of what to do to meet unmet needs, are mainly unconscious. Since they are encoded in the implicit memory system, making them subject to revision is a lengthy process [4] requiring intensive therapy that works at the level of affect, e.g., psychodynamic treatment.

3. Most behavioral sequences aimed at meeting our needs are executed unconsciously. Cognition is mostly unconscious—roughly 95% of goal-directed activities are executed unconsciously [9]. Both our patients and we, as clinicians, place undue faith in the ability of our prefrontal cortex to consciously problem-solve through top-down regulation of our primary emotions. How often have our patients said, ‘Doctor if I can just understand (myself/my past) then I will be able to change my behaviour’?

It is relevant to our clinical work to acknowledge that behaviours are predictions to meet the needs of the BES. Friston [7] offers a contemporary definition of psychopathology, arguing that “if psychology—read as belief updating in the brain—can be cast as a computational process of inference, it follows that *psychopathology just is false inference*”. Therefore, individuals with psychiatric disorders perform false inferences (i.e., predictions) in an enduring way, in one or more areas of their mental functioning. And due to these false inferences, they behave in ways that fail to meet their emotional needs.

## Clinical Application of Affective Neuroscience to Psychiatry 

A neuropsychoanalytic understanding of the biological function of emotion and how it influences behaviour contributes to psychiatric assessment and management [10].

### Assessment

*Formulation:* Rather than focusing on the patient’s symptoms, it may be preferable to think more causally, i.e., what are the unmet emotional needs and false inferences (predictions) giving rise to these symptoms? This approach may also advance the biopsychosocial model, which has been critiqued for being rather eclectic, vague, and generic [11]. A Neuropsychoanalytic approach to the formulation may offer a more connected and sequenced conceptualization of the patient’s presentation by asking: (1) what is the unmet need (2) what is the individual doing to meet this need, and (3) what are his ensuing behaviours (i.e., his defenses) to deal with the consequences of the ongoing unmet emotional need? While these are the questions Solms [4] addresses in his psychoanalytic work, they have relevance in other clinical settings.

*Diagnosis:* Applying BES may refine the discourse on conditions like personality disorder [12], as it carves at the biological joints more than the DSM-5/ICD11.

### Management

Is our treatment definitive or symptomatic? If we view affect as the signal of unmet need, then prescribing medication may be seen as working at a symptomatic level. While this may be necessary, we need to recognize that this is not a definitive treatment—which necessitates addressing the false inference, such as the pathogenic prediction the patient is making to meet the needs of the Basic Emotional System [13]. These are reasons why health service planning needs to include psychodynamic psychotherapy—it is necessary as a treatment modality that is long-term and works at the level of affect.

## Conclusion 

The contribution Neuropsychoanalysis can make regarding, e.g., assessment, management, and expectations of interventions (both for patients and ourselves) can lead to a fundamental shift in our practice as psychiatrists, and thus has significant implications for the training of psychiatrists at both the trainee and consultant levels.
